# Choosing ℓ^*p*^ norms in high-dimensional spaces based on hub analysis

**DOI:** 10.1016/j.neucom.2014.11.084

**Published:** 2015-12-02

**Authors:** Arthur Flexer, Dominik Schnitzer

**Affiliations:** Austrian Research Institute for Artificial Intelligence, Freyung 6/6, Vienna, Austria

**Keywords:** High-dimensional data analysis, Hubness, Fractional norms, Concentration of distances

## Abstract

The hubness phenomenon is a recently discovered aspect of the curse of dimensionality. Hub objects have a small distance to an exceptionally large number of data points while anti-hubs lie far from all other data points. A closely related problem is the concentration of distances in high-dimensional spaces. Previous work has already advocated the use of fractional ℓ^*p*^ norms instead of the ubiquitous Euclidean norm to avoid the negative effects of distance concentration. However, which exact fractional norm to use is a largely unsolved problem. The contribution of this work is an empirical analysis of the relation of different ℓ^*p*^ norms and hubness. We propose an unsupervised approach for choosing an ℓ^*p*^ norm which minimizes hubs while simultaneously maximizing nearest neighbor classification. Our approach is evaluated on seven high-dimensional data sets and compared to three approaches that re-scale distances to avoid hubness.

## 1 Introduction

A number of publications [1–3] have recently focused on the emergence of hubs as a new aspect of the curse of dimensionality [4], a term which refers to challenges due to high dimensionality of data spaces. Hubs have an exceptionally low distance to a high number of objects and therefore are nearest neighbors of an exceptionally large percentage of data points. As a result, other objects (anti-hubs) are pushed out of all nearest neighbor lists. It was shown that this behavior has a negative impact on many machine learning tasks including classification [1], nearest neighbor based recommendation [5], outlier detection [1,6] and clustering [7]. Affected areas of application include multimedia retrieval [8], collaborative filtering [9,10], speaker verification [11] and speech recognition [12].

A closely related phenomenon is the concentration of distances in high dimensional spaces. The concentration effect is the surprising characteristic of all points in a high dimensional space to be at almost the same distance to all other points in that space. Already in some of the publications establishing the property of concentration of distances [13], it has been argued that fractional norms (ℓ^*p*^ norms where p<1) might be able to mitigate the phenomenon. The authors were able to show that concentration of distances can be reduced by using fractional norms, but deciding which exact norm to use is not straight forward. This result is the motivation for us to examine the relation of different ℓ^*p*^ norms and hubness in high-dimensional spaces. Our work pursues the idea of choosing an ℓ^*p*^ norm to counter problems in high-dimensional data spaces in the light of the effects of hubs and anti-hubs. We show empirically that the degree of hubs and anti-hubs in a data set can help selecting the optimum ℓ^*p*^ norm. Based on these results we propose a fully unsupervised approach for choosing an ℓ^*p*^ norm which maximizes nearest neighbor classification. This approach is compared to three methods that re-scale distances in order to reduce hubness. We first review related work in Section 2, then present our approach for finding an ℓ^*p*^ norm as well as the three re-scaling methods in Section 3, describe the seven data sets used for evaluation in Section 4, present all results in Section 5 and conclude in Section 6. This work is an expanded version of a conference publication [14], with the comparison to re-scaling methods being the main extension.

## 2 Related work

In our review of related work, we first outline the relation between the phenomenon of hubness and concentration of distances. Next we review existing work on choosing ℓ^*p*^ norms that are able to reduce the concentration effect. At last we review work on re-scaling distance spaces to secondary distance measures which are able to reduce hubness in high-dimensional data spaces.

As is common practice in explaining the hubness problem [1], we first give a short review of the closely related phenomenon of concentration of distances in high-dimensional data spaces. Concentration is the fact that all points are at almost the same distance to each other in a high-dimensional space [15]. It is usually measured as a ratio between spread and magnitude, e.g. the ratio between the standard deviation of all distances to an arbitrary reference point and the mean of these distances. If the standard deviation stays constant with increasing dimensionality while the mean keeps growing, the ratio converges to zero with dimensionality going to infinity. In such a case it is said that the distances concentrate. Proofs concerning concentration of distances and all points being at the same distance to all other points have been formulated for dimensionality approaching infinity. Radovanović et al. [1] presented the argument that for any finite dimensionality, some points are expected to be closer to the center of all data^1^ than other points and are at the same time closer, on average, to all other points. Such points closer to the center have a high probability of being hubs, i.e. of appearing in nearest neighbor lists of many other points. Points which are further away from the center have a high probability of being ‘anti-hubs’, i.e. points that never appear in any nearest neighbor list. This was evaluated [1] for cosine and Euclidean (ℓ^2^) norm on real world data but also observed for $\ell^{0.5}$ using i.i.d. normal and uniform data. It is also important to note that the degree of concentration and hubness is linked to the intrinsic rather than extrinsic dimension of the data space. Whereas the extrinsic dimension is the actual number of dimensions of a data space the intrinsic dimension is the, often much smaller, number of degrees of freedom of the submanifold in which the data space can be represented [15]. Previous research [3] has shown that real world data with extrinsic dimensionality as small as 34 can already exhibit the negative effects of hubness.

The concentration effect was studied by Aggarwal et al. [13] for Euclidean and fractional ℓ^*p*^ norms. The Euclidean norm is part of the family of Minkowski norms:

(1) $$D_{x,y}={\left(\underset{i}{\sum }{|(x_{i}-y_{i})|}^{p}\right)}^{1/p}$$

When *p*=2, the Minkowski norm corresponds to the Euclidean norm, *p*=1 defines the Manhattan or city-block metric. Minkowski norms with p<1 are called fractional norms. Note that for 0<p<1 the triangle inequality does not hold and therefore fractional norms are sometimes called prenorms [15] or quasi-norms. Aggarwal et al. [13] come to the conclusion that from a theoretical and empirical perspective, the Euclidean (ℓ^2^) norm is often not the preferred metric for high-dimensional data mining applications since fractional norms are less prone to distance concentration. More specifically, the authors showed that all ℓ^*p*^ norms concentrate, but the degree of concentration depends on both the distribution of the high-dimensional data and the value of *p*. This dependency on the data distribution has recently been explored in more detail [16,17]. Experiments [15] also show that choosing the right fractional norm, as opposed to the Euclidean norm, could significantly improve the effectiveness of standard *k*–nearest neighbor (*k*NN) classification in high-dimensional spaces. This observation was more closely investigated by François et al. [18] who follow a supervised approach to infer the optimum ℓ^*p*^ norm using labeled training data. More precisely, the authors use a simple regression model to choose an optimal norm which is then evaluated on more elaborate regression models.

To avoid this problem of concentration of distances the use of ‘Shared Neighbor Distances’ has been proposed by Houle et al. [19], who raised the question whether these secondary distance measures are able to “defeat the curse of dimensionality”. ‘Shared nearest neighbors’ (SNN) was first proposed as a similarity measure by Jarvis and Patrick [20] to improve the clustering of ‘non-globular’ clusters. As the name suggests, SNN similarity is based on computing the overlap between the *k* nearest neighbors of two objects and therefore only uses rank and not distance information. Houle et al. [19] argued that the rank information SNN is based on might still be meaningful even when distances concentrate in high dimensions. In an extensive study using artificial and three real world image recognition data sets, the authors show that SNN is indeed able to reduce the concentration of distances. The secondary SNN distances also result in improved image classification rates measured as area under receiver operating curve based on nearest neighbor classification. But the authors do not make a connection to the hubness phenomenon which at the time of their study was not very well-known.

Two methods (local scaling (LS) and mutual proximity (MP)), which are somewhat related to SNN, have been proposed by Schnitzer et al. [3] as a way to reduce the negative effects of hubness. Both methods aim at repairing asymmetric nearest neighbor relations. The asymmetric relations are a direct consequence of the presence of hubs since a hub *y* is the nearest neighbor of *x*, but the nearest neighbor of the hub *y* is another point *a* (a≠x). This is because hubs are by definition nearest neighbors to very many data points but only a fixed number of data points can be the *k*-nearest neighbors to a hub. Both methods re-scale distances and return a small distance between two objects only if their nearest neighbors concur. Whereas LS uses local distance information to achieve this, MP is based on probability distribution models of the full distance space. The positive impact of LS and MP was measured as a decrease of hubness and an accuracy increase in *k*-nearest neighbor classification experiments on 30 real world data sets. Both methods LS and MP have already been compared directly to SNN by Flexer et al. [21]. It was shown that SNN does reduce hubness, but less than LS and MP, and that it is only able to improve classification accuracy for half of the six data sets used in the study.

The so-called ‘hubness-aware’ SNN approaches have been studied for nearest neighbor classification [22] and clustering [7] by Tomašev et al. These hubness-aware approaches are based on the notion of ‘bad hubs’, i.e. hubs that show a disagreement of class information for the majority of data points they are nearest neighbors to. A quantitative index for the ‘bad hubness’ of a data point can be used for a weighting scheme in *k*-nearest neighbor classification [1,22]. These hubness-aware SNN approaches use class label information to compute secondary measures and are therefore less general than the fully unsupervised approaches like classic SNN, LS or MP. Classic SNN has been compared to hubness-aware SNN on a number of artificial data sets and within an image recognition context [22]. Both types of SNN approaches are able to reduce hubness and improve nearest neighbor classification, with hubness-aware SNN being better at classification, which seems as expected since it does use class label information.

## 3 Methods

We now present our method to choose an ℓ^*p*^ norm based on hubness analysis. We also review three methods that reduce hubness by re-scaling distance matrices and computing secondary distance measures. All four methods are unsupervised and use class label information only during evaluation. Like Aggarwal et al. [13] we will evaluate the impact of changing the ℓ^*p*^ norm and of re-scaling the distances by reporting the *k*NN classification accuracy using leave-one-out cross-validation. The classification is performed via a majority vote among the *k* nearest neighbors, with the class of the nearest neighbor used for breaking ties. We denote the *k*NN accuracy as *C*^*k*^. Since data objects within a class are supposed to be more similar to each other than to objects from other classes, higher classification accuracy indicates better distance measures.

To test for statistical significance of differences in classification accuracy we use McNemar׳s test (see [23] and [24] for a discussion of using this test in conjunction with leave-one-out classification). When comparing two algorithms A and B, only classification instances where A and B disagree are being analyzed. More specifically, it is tested whether the number of times that A classifies correctly and B does not is significantly different from the number of times B classifies correctly and A does not.

### 3.1 Choosing an ℓ^*p*^ norm

To choose a norm based on hubness analysis, we first need to identify hubs and anti-hubs by looking at all NN lists of a data set *X*. For a given neighborhood size *n*, the *n*-occurrence ($O^{n}(x)$) of a point x∈X is then computed by counting the number of occurrences of *x* in the NN of each point $x_{i}\in X,x_{i}\neq x$. Using *O*^*n*^ we then define the set of hubs (*H*^*n*^) and anti-hubs (*A*^*n*^) as

(2) $$A^{n}=\{a\in X|O^{n}(x)=0\}$$

(3) $$H^{n}=\{h\in X|O^{n}(x)\geq 2n\}$$

Anti-hubs (a) never occur in the NN, i.e. have a *O*^*n*^ of zero, while hubs (h) occur at least twice as often (2*n*) as expected. To asses the overall impact of hubness in a data set Radovanović et al. [1] proposed to compute ‘hubness’ (*S*^*s*^), which he defined as the skewness of the histogram of the *O*^*s*^. The higher the measured sample skewness of the *O*^*s*^ histogram, the higher the impact of hubs in the NN^2^:

(4) $$S^{s}=\frac{E[{\left(O^{s}-\mu_{O^{s}}\right)}^{3}]}{\sigma_{O^{s}}^{3}}.$$

We use this measure to identify high-dimensional data sets showing strong hubness in the Euclidean space by choosing data sets where $S^{s=5}>2$. Full detail on these high dimensional data sets is given in Section 4.

To measure the impact of hubs and anti-hubs on a given data set we propose two measures: (i) anti-hub occurrence (*A*^*n*^_*occ*_) and (ii) hub occurrence (*H*^*n*^_*occ*_). Whereas *A*^*n*^_*occ*_ is the percentage of data points that act as anti-hubs, *H*^*n*^_*occ*_ is the percentage of hub points in all NN lists. We use these measures in our experiments to evaluate a given ℓ^*p*^ norm in terms of anti-hubs and hubs at a selected neighborhood radius *n*:

(5) $$A_{\mathrm{occ}}^{n}=\frac{1}{\left|X\right|}|A^{n}|$$

(6) $$H_{\mathrm{occ}}^{n}=\frac{1}{\left|X\right|}\underset{h\in H^{n}}{\sum }\frac{O^{n}(h)}{n}$$

We choose the ℓ^*p*^ norm where the corresponding anti-hub occurrence (*A*^*n*^_*occ*_) or hub occurrence (*H*^*n*^_*occ*_) is minimal.

We do not use the hubness measure (*S*^*s*^, i.e. the skewness of the *O*^*s*^) for this purpose since it does not equally account for hubs and anti-hubs in the measurements. By computing the sample skewness, hubs with a theoretical maximum $O^{s}(h)=|X|-1$ have a much higher influence on the measure than anti-hubs since their difference to the *μ*_*O*_^*s*^ contributes to *S*^*s*^ to the third power. Additionally our experiments with *S*^*s*^ in this context did not show a smooth but oscillating change of values when stepping through different ℓ^*p*^ norms, making *S*^*s*^ unfit for our purpose.

### 3.2 Computing secondary measures

The following three methods compute secondary distance measures and have already been shown [3,21] to reduce hubness. As described in Section 2, all three approaches try to symmetrize nearest neighbor relations. They will be compared to our method for choosing an ℓ^*p*^ norm in Section 5.2.

*Shared Nearest Neighbors* (*SNN*): SNN is based on rank information of distances and is computed as a set of intersection of the nearest neighbor lists NN of size *r* of two objects *x*, *y*:

(7) SNN(x,y)=|NN(x)∩NN(y)|/r.

This way SNN strictly strengthens symmetric nearest neighbor relations which in turn leads to a reduction of hubness. Since our previous research [21] (using three of the same data sets as in this paper) has shown that NN lists larger than 10 did not really improve results, we use SNN with *r*=10.

*Local Scaling* (*LS*): Local scaling [25] transforms arbitrary distances to the so-called *affinities* (that is, similarities) according to

(8) $$\mathrm{LS}(D_{x,y})=\exp(-\frac{D_{x,y}^{2}}{\sigma_{x}\sigma_{y}}),$$

where *σ*_*x*_ denotes the distance between object *x* and its *q*׳th nearest neighbor. $\mathrm{LS}(D_{x,y})$ tends to make neighborhood relations more symmetric by including local distance statistics of both data points *x* and *y* in the scaling. We use LS with *q*=10, as it returned the best and most stable results. This variant of LS is identical to the one used in [21] including the parameter choice for *q*.

*Mutual Proximity* (*MP*): MP reinterprets the original distance space so that two objects sharing similar nearest neighbors are more closely tied to each other, while two objects with dissimilar neighborhoods are repelled from each other. This is done by transforming the distance of two objects into a mutual proximity in terms of their distribution of distances. It was shown that by using this mutual reinterpretation of distances hubness is decisively reduced, while the intrinsic dimensionality of the data stays the same [3]. To compute MP, we assume that the distances $D_{x,i=1‥m}$ from an object *x* to all other objects in our data set follow a certain probability distribution. Therefore any distance $D_{x,y}$ can be reinterpreted as the probability of *y* being the nearest neighbor of *x*, given their distance $D_{x,y}$ and the probability distribution *P*(*X*). In this work we use the empirical distribution for all experiments. MP is defined as the probability that *y* is the nearest neighbor of *x* given *P*(*X*) and *x* is the nearest neighbor of *y* given *P*(*Y*):

(9) $$\mathrm{MP}(D_{x,y})=P(X>D_{x,y}\cap Y>D_{y,x}).$$

Computing 1−SNN,1−LSand1−MP turns the similarities into distance measures.

## 4 Data

We use the hubness measure *S*^*s*^ (see Section 3.1) to identify high-dimensional data sets showing strong hubness in the Euclidean space by choosing data sets where $S^{s=5}>2$. The data sets identified are *Protein*, *Splice*, *Gisette* and *Dexter* from the UCI machine learning archive [26], two standard image-classification data sets (*Leeds Butterfly* [27], 17 *Flowers* [28]) and a data set from the text-retrieval domain, *Twitter* (*C1ka*) [29]. The dimensionality *d*, size of data set *m*, number of classes *c* and hubness $S^{s=5}$ of the original Euclidean space are listed in Table 1. Data sets are used as they are available on their respective websites without any additional normalization. The extrinsic dimensionality ranges from 60 (*Splice*) to 49 820 (*Twitter* (*C1ka*)), while the measured hubness ranges from rather moderate values of 2.9 (*Gisette* and *Dexter*) to extreme values of 43.1 (*Protein*) in ℓ^2^.

## 5 Experiments and results

We will now evaluate in Section 5.1 whether our proposed method is able to find ℓ^*p*^ norms which perform better than standard ℓ^2^ norms. Then we will compare these results in Section 5.2 to those obtained with re-scaling methods described in Section 3.2.

### 5.1 Choosing an ℓ^*p*^ norm

**Fig. 1:**
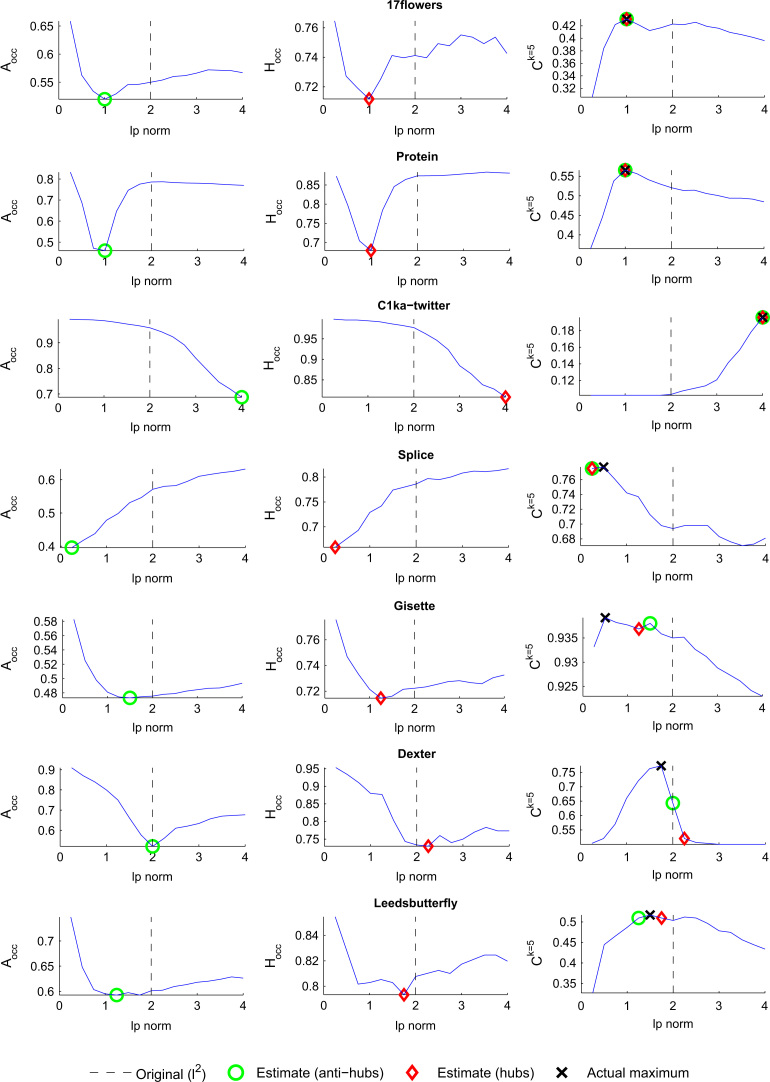
The minimum in anti-hub (*A*^*n*^_*occ*_) and hub (*H*^*n*^_*occ*_) occurrence while changing the ℓ^*p*^ norm is closely related to the maximum *k*NN classification rate (*C*^*k*^). See Section 5.1.

To investigate the relation of hubs and anti-hubs to a certain ℓ^*p*^ norm we compute *A*^*n*^_*occ*_ and *H*^*n*^_*occ*_ (see Eqs. (5) and (6)) for our selected data sets. We set our neighborhood size to *n*=1 (i.e., we only look at each point׳s nearest neighbor) while changing the ℓ^*p*^ norm from p=0.25,0.5,0.75,…,4. For each step in *p* we compute the *k*NN classification rate $C^{k=5}$. Fig. 1 plots the results for each of the selected data sets. *A*^*n*^_*occ*_ is plotted in the first column of the figures, *H*^*n*^_*occ*_ in the second column and the classification rate $C^{k=5}$ in the third column of the figures. Each of the measures is computed while varying parameter *p* as discussed. Note that results using a larger neighborhood size to compute *A*^*n*^_*occ*_ and *H*^*n*^_*occ*_ or with one nearest neighbor classification ($C^{k=1}$) did not substantially change the following results.

Looking at the figures we first note a very high similarity between the anti-hub (*A*^*n*^_*occ*_) and hub (*H*^*n*^_*occ*_) curves. This behavior is as expected since a higher number of objects not occurring in the NN lists at all have to lead to higher *O*^*n*^ values for the remaining objects. In addition, the *k*NN classification accuracy (*C*^*k*^) results are highest at values of *p* different from 2, which is in accordance with results reported by Aggarwal et al. [13]. Furthermore the peak in *C*^*k*^ concurs with either *A*^*n*^_*occ*_ or *H*^*n*^_*occ*_ being at or close to their minimum. In view of the fact that neither the computation of *A*^*n*^_*occ*_ nor *H*^*n*^_*occ*_ include any class label information, these empirical results give a strong argument that both measures could be effective for choosing the optimum ℓ^*p*^ norm.

Table 1 summarizes the results. In the table we list the *original k*NN classification rate (*C*^*k*^) in ℓ^2^, the actual maximum (*max* *C*^*k*^) and the two estimated maxima using *A*^*n*^_*occ*_ and *H*^*n*^_*occ*_. In three data sets (*17 Flowers*, *Protein* and *Twitter* (*C1ka*)) we are able to identify the best ℓ^*p*^ norm according to *C*^*k*^ by using the minima of both *A*^*n*^_*occ*_ or *H*^*n*^_*occ*_. The increase in *C*^*k*^ ranges from 0.9 to 9.3 percentage points. The optimum norm is twice ℓ^1^ and once ℓ^4^. In three further cases (*Splice*, *Gisette* and *Leeds Butterfly*) both measures are able to identify a better ℓ^*p*^ norm than the Euclidean base case, but closely fail to identify the actual maximum. The increase in *C*^*k*^ ranges from 0.4 to 8.1 percentage points. In the case of *Dexter* and by using *H*^*n*^_*occ*_ (*p*=2.25) as decision, the proposed method would lead to a drop in classification accuracy by 12.3 percentage points. Using *A*^*n*^_*occ*_ however would stay with the Euclidean norm, thus suggesting no change of norm. The actual maximum is at *p*=1.75. Upon closer inspection of the results, we see *H*^*n*^_*occ*_ closely missed ℓ^2^ because a single hub occurrence ($O^{n}(h)$) is increased by a count of 1 (and the actual *C*^*k*^ maximum is missed due to an increase of 4 counts). The small data set size (|X|=300) could be the cause for this result. Note that discussion of statistical significance of results is provided in Section 5.2.

To sum up the results, we like to state that (i) for all seven data sets the optimum value for *p* is different from 2, (ii) it is possible to find an ℓ^*p*^ norm that is better than ℓ^2^ in six out of seven cases based on hubness analysis and (iii) in three out of seven cases we are able to find the actual optimal norm.

### 5.2 Comparison to secondary measures

**Fig. 2:**
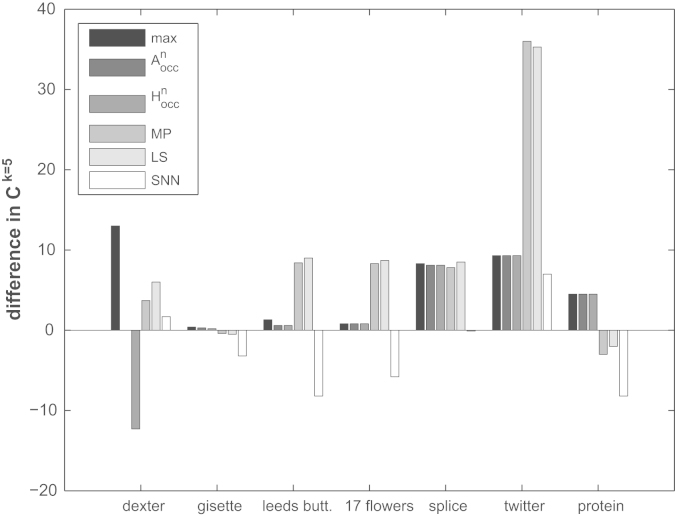
Comparison of classification accuracy results $C^{k=5}$ on the *y*-axis for all seven data sets (*x*-axis) and all six methods depicted as six bars per data set. Shown are differences in absolute percentage points relative to using the original ℓ^2^ norm.

We now compare the results for finding optimal ℓ^*p*^ norms based on hubness analysis reported in Section 5.1 to results achieved by using secondary distance measures. We report *k*NN classification rates $C^{k=5}$ based on mutual proximity (MP), local scaling (LS) and shared nearest neighbors (SNN) for all data sets in Table 2. We also give classification results for the original Euclidean space (*orig*), actual maximum (max) and estimated maximum using *A*^*n*^_*occ*_ or *H*^*n*^_*occ*_. The corresponding differences in absolute percentage points relative to using the original ℓ^2^ norm are shown in Fig. 2 as a bar graph. The top performing approach for each of the seven data sets is printed in bold in Table 2. Every result that is statistically significantly better than the corresponding result achieved for Euclidean (ℓ^2^) distances is marked with an asterisk. It can be seen that for none of the data sets the original distance space based on the ℓ^2^ norm is the best. There always exist superior alternatives which result in significantly higher classification accuracy. For four data sets (*Leeds Butterfly*, *17 flowers*, *Splice*, *Twitter*) one of the secondary measure approaches performs best (three times *LS*, one time *MP*). The gain in accuracy compared to the best performing ℓ^*p*^ norm ranges from 2.2 (*Splice*) to 26.7 (*Twitter*) percentage points. For two data sets (*Protein* and *Gisette*) both hubness based approaches (*A*^*n*^_*occ*_ and *H*^*n*^_*occ*_) work better than any of the secondary distance approaches. For data set *Dexter* the theoretically optimal ℓ^*p*^ norm outperforms all other approaches including the ones based on secondary measures. As has already been observed [21], the *SNN* approach performs worse on all data sets when compared to *MP* and *LS*. The last line in Table 2 gives the average gain in absolute percentage points relative to using the original ℓ^2^ norm (average taken across all seven data sets). As can be seen, all methods except SNN are able to improve results on average. The best overall performers are MP and LS.

To sum up the results, it seems to be highly problem dependent whether an ℓ^*p*^ norm obtained via hubness analysis or re-scaled secondary measures perform best.

## 6 Conclusion

This work linked finding the optimum ℓ^*p*^ norm (in terms of *k*NN classification rates) to hubs and anti-hubs occurring in high-dimensional data. In an empirical study we presented strong evidence that the optimum ℓ^*p*^ norm for data sets with high hubness in the Euclidean space can be found at values of *p*, where hubs and anti-hubs have their minimal impact on the data. To identify these points we propose to measure the hub (*H*^*n*^_*occ*_) or anti-hub (*A*^*n*^_*occ*_) occurrence as defined in this work. Using these measures we were able to identify better norms in six of the seven analyzed data sets. Comparison to three methods that re-scale distances to avoid negative effects of hubness showed that the choice of an optimal distance function is highly problem dependent. For four out of the seven data sets secondary distance measures even further improve results when compared to our approach of choosing an ℓ^*p*^ norm. But it is also evident that for all of the seven high-dimensional data sets in our empirical evaluation there always exist more optimal alternatives to the standard Euclidean distance.

Future work will analyze the relation of different ℓ^*p*^ norms and the concentration of distances in real world data. This could also further illuminate the relation between hubness and concentration. Another interesting point is to research the impact of using different ℓ^*p*^ norms in the context of classifiers beyond simple *k*NN classification.

## Figures and Tables

**Table 1:** Data sets, their dimensionality *d* and size *m*, number of classes *c*, hubness ($S^{s=5}$), classification rates (*C*^*k*^) in the original Euclidean space $\left(\ell^{2}\right)$, actual maximum (max *C*^*k*^) and estimated maximum ℓ^*p*^ based on anti-hubs *A*^*n*^_*occ*_*est* and hubs *H*^*n*^_*occ*_*est*. Better or equal *C*^*k*^ when compared to the original data are given in bold, an asterisk indicates that respective methods were able to find the actual maximum.

*Data set*	*d*	*m*	*c*	$S^{s=5}$	*Original*		max*C*^*k*^		*A*^*n*^_*occ*_*est*		*H*^*n*^_*occ*_*est*	
					ℓ^*p*^	$C^{k=5}$ (%)	ℓ^*p*^	$C^{k=5}$ (%)	ℓ^*p*^	$C^{k=5}$ (%)	ℓ^*p*^	$C^{k=5}$ (%)
Dexter	20 000	300	2	2.9	2	64.3	1.75	**77.3**	2	**64.3**	2.25	52.0
Gisette	5000	6000	2	2.9	2	93.5	0.5	**93.9**	1.5	**93.8**	1.25	**93.7**
Leeds Butt.	36 000	832	10	3.5	2	50.4	1.5	**51.7**	1.25	**51.0**	1.75	**51.0**
17 Flowers	36 000	1360	17	3.9	2	42.3	1	**43.1**	1	^⁎^**43.1**	1	^⁎^**43.1**
Splice	60	1000	2	5.6	2	69.4	0.5	**77.7**	0.25	**77.5**	0.25	**77.5**
Twitter	49 820	969	17	14.6	2	10.3	4	**19.6**	4	^⁎^**19.6**	4	^⁎^**19.6**
Protein	357	6621	3	43.1	2	52.1	1	**56.6**	1	^⁎^**56.6**	1	^⁎^**56.6**

**Table 2:** Data sets, classification rates ($C^{k=5}$) in percent in the original Euclidean space (*orig*), actual maximum (max *C*^*k*^), estimated maximum based on anti-hubs *A*^*n*^_*occ*_*est* and hubs *H*^*n*^_*occ*_*est*, classification rates based on secondary measures computed with *MP*, *LS* and *SNN*. Best classification results $C^{k=5}$ per data set printed in bold. Classification results which are significantly better than the ones achieved in the original Euclidean space are marked with an asterisk (McNemar test, 5% significance level, degrees of freedom =1). The last line gives the average gain in absolute percentage points relative to using the original ℓ^2^ norm.

Data set	Orig	max *C*^*k*^	*A*^*n*^_*occ*_*est*	*H*^*n*^_*occ*_*est*	MP	LS	SNN
Dexter	64.3	**77.3**^⁎^	64.3	52.0	68.0	70.3^⁎^	66.0
Gisette	93.5	**93.9**^⁎^	93.8^⁎^	93.7	93.1	93.0	90.3
Leeds Butt.	50.4	51.7	51.0	51.0	58.8^⁎^	**59.4**^⁎^	42.2
17 Flowers	42.3	43.1	43.1	43.1	50.6^⁎^	**51.0**^⁎^	36.5
Splice	69.4	77.7^⁎^	77.5^⁎^	77.5^⁎^	77.2^⁎^	**77.9**^⁎^	69.3
Twitter	10.3	19.6^⁎^	19.6^⁎^	19.6^⁎^	**46.3**^⁎^	45.6^⁎^	17.3^⁎^
Protein	52.1	**56.6**^⁎^	**56.6**^⁎^	**56.6**^⁎^	49.1	50.1	43.9
Ave. gain	–	5.37	3.37	1.6	8.69	9.29	−2.40

## Footnotes

1 Note that in case of multimodal data distributions, hubs tend to be close to centers of individual components.

2 Methods for hubness data analysis are available in our Matlab hub–toolbox: http://www.ofai.at/research/impml/projects/hubology.html.
